# 

**Published:** 1895-03-16

**Authors:** 


PRICE TWOPENCE.

No. 442, Vol. XVII,—March 16th, 1895.

[Registered at the General Post Office as a Newspaper.

WITH EXTRA NURSING SUPPLEMENT.

Per Annum, 10s. 6d., post free.

Monthly Parts, 6d.; post free, 8d.

Ditto per Annum, 8s. 6d., post free.

### THE HOSPITAL

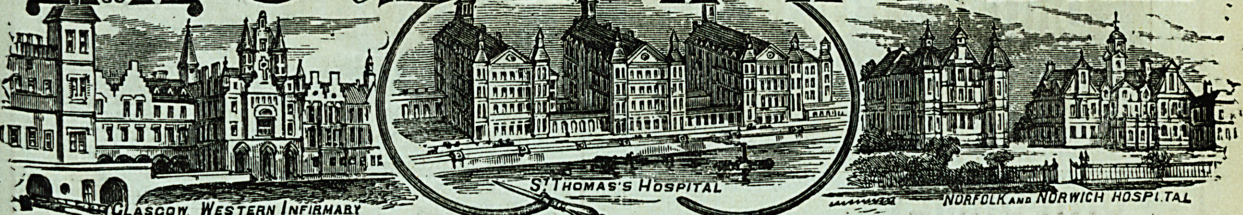

No. 442. Vol. XVII. WITH EXTRA NURSING SUPPLEMENT. SATURDAY, MARCH 16TH, 1895.

